# Inhibition of Propofol Anesthesia on Functional Connectivity between LFPs in PFC during Rat Working Memory Task

**DOI:** 10.1371/journal.pone.0083653

**Published:** 2013-12-27

**Authors:** Xinyu Xu, Yu Tian, Shuangyan Li, Yize Li, Guolin Wang, Xin Tian

**Affiliations:** 1 School of Biomedical Engineering, Tianjin Medical University, Tianjin, China; 2 Department of Anesthesiology, Tianjin Medical University General Hospital, Tianjin, China; Massachusetts General Hospital, United States of America

## Abstract

Working memory (WM) refers to the temporary storage and manipulation of information necessary for performance of complex cognitive tasks. There is a growing interest in whether and how propofol anesthesia inhibits WM function. The aim of this study is to investigate the possible inhibition mechanism of propofol anesthesia based on the functional connections of multi-local field potentials (LFPs) and behavior during WM tasks. Adult SD rats were randomly divided into 3 groups: pro group (0.5 mg·kg^−1^·min^−1^,2 h), PRO group (0.9 mg·kg^−1^·min^−1^, 2 h) and control group. The experimental data were 16-channel LFPs obtained at prefrontal cortex with implanted microelectrode array in SD rats during WM tasks in Y-maze at 24, 48, 72, 96, 120 hours (day 1-day 5) after propofol anesthesia, and the behavior results of WM were recoded at the same time. Directed transfer function (DTF) method was applied to analyze the connections among LFPs directly. Furthermore, the causal networks were identified by DTF. The clustering coefficient (*C*), network density (*D*) and global efficiency (*E_global_*) were selected to describe the functional connectivity quantitatively. The results show that: comparing with the control group, the LFPs functional connectivity in pro group were no significantly difference (*p*>0.05); the connectivity in PRO group were significantly decreased (*p*<0.05 at 24 hours, *p*<0.05 at 48 hours), while no significant difference at 72, 96 and 120 hours for rats (*p*>0.05), which were consistent with the behavior results. These findings could lead to improved understanding the mechanism of inhibition of anesthesia on WM functions from the view of connections among LFPs.

## Introduction

Propofol (2, 6-diisopropylphenol) is an intravenous anesthetic that has been used for induction and maintenance of anesthesia in clinical practice. In recent years there are some research reports about memory dysfunction caused by propofol anesthesia, especially in elderly and pediatric patients[1]–[4]. This phenomenon has also been observed in animal models. Propofol anesthesia decreased hippocampal cell proliferation and produced learning impairment in young rats [5]. The inhibition of long-term potentiation (LTP) in the hippocampus has been attributed to the amnesic effect of propofol [6]. A short-term propofol anesthesia of approximately 4.5 h duration induced histological neurodegeneration in the immature rat brain and led to persistent learning deficits [7]. However, the mechanisms of memory impairment induced by propofol remain unclear. Brain functional connectivity network analysis may render a new method to research the problem [8].

Brain is a formidably complicated network. Functional connectivity is often defined as the dynamic emergence of coherent physiological activity (for example, phase-locked high-frequency electromagnetic oscillations) that can span the multiple spatially distinct brain regions [9], [10]. Brain functional connectivity networks are thought to provide the physiological basis for information processing and mental representations [11]–[13]. Analysis of brain functional connectivity networks based on graph theory applies a new method useful for the studies in research of brain cognitive function [14], [15] and mental or neurological disease [16]–[18]. Network-based analyses of brain functional connectivity have been used to describe normal cognitive functions including attention [19], memory [20]–[23] and learning [24], [25]: the posterior parietal cortex during tasks involving autobiographical memory and self-referential processes [26], the medial prefrontal cortex in social cognitive processes [27], the medial temporal-lobe in episodic memory [28], and the angular gyrus in semantic processing [29]. Network-based analyses of brain functional connectivity have also been a powerful tool to understand the dysfunction of psychiatric and neurological disorders. Koenig T et al. applied the method to different frequency bands EEG data and confirmed the hypothesis of functional disconnection of neuro-cognitive networks in patients with mild cognitive impairment and Alzheimer Dementia [30]. Uhlhaas PJ et al. summarized that schizophrenia involved abnormal functional connectivity network which was related to cognitive dysfunctions [31], [32]. Murias M et al. applied the method to theta frequency range and alpha range EEG data acquired from autism spectrum disorder subjects in the eyes closed resting state, and found that robust patterns of over- and under-connectivity were apparent at distinct spatial and temporal scales [33]. Dejean C et al. investigated the functional connectivity network among LFPs in the cortex acquired from freely moving rats with Parkinson's disease, and confirmed that neuronal signal transmission in the functional connectivity network was altered after dopamine depletion [34].

Neural signals used to establish functional connectivity network compose of functional magnetic resonance imaging (fMRI), electroencephalogram (EEG), magnetoencephalogram (MEG) and local field potentials (LFPs) [35]. fMRI has good spatial resolution but poor temporal resolution, and fMRI measures activation-related haemodynamics rather than neuronal activity; EEG and MEG have better temporal resolution, but they often have worse spatial resolution than fMRI; while LFP was recorded by multi-channel micro-electrode array, measures neuronal activity directly and has better spatial and temporal resolution [36].

Working memory (WM) refers to the temporary storage and manipulation of information necessary for performance of complex cognitive tasks [37], [38]. WM performance was uniquely correlated with power in the theta and gamma frequency, and gamma power was increased by WM training [39]. Prefrontal cortex (PFC) participates in WM, and plays an active role in the shift of attention [40] and task switching [41].

The above evidences converge to a theory that, functional connectivity of causal network among specific frequency bands of LFPs may serve as an important the neurobiological mechanism underlying the effect of propofol on WM function. In this study, we investigated whether the inhibition of propofol on WM was dose-dependent, the possible inhibition mechanism of propofol anesthesia based on the functional connections of multi-local field potentials (LFPs) and behavior during WM tasks at 24, 48, 72, 96, 120 hours (day 1- day 5) after propofol anesthesia.

## Materials and Methods

### Ethics Statement

This study was approved by the Institutional Animal Care and Use Committee of Tianjin Medical University under the NIH Guide for the Care and Use of Laboratory Animals.

### Subjects and housing conditions

A total of 21 male Sprague-Dawley rats (approximately 3mo), purchased from Institute of Radiation Medicine Chinese Academy of Medical Sciences, were housed in standard shoebox cages, had free access to drinking water, and were kept under a 12-hr light–dark cycle. The rats' care and surgical procedures were in accordance with the National Institutes of Health Guide for the Care and Use of Laboratory Animals (1997) and with the Tianjin medical University guidelines for the use and care of laboratory animals in research.

### Chronic implant surgery

The surgical procedure has already been described in detail [42]. All SD rats were implanted with a 16-channel microelectrode array (30 µm diameter wires with 600–800 kΩ impedance, arranged in a 2×8 grid with 200-µm spacing between wires) in the PFC under chloral hydrate anesthesia (350 mg/kg). The following coordinates (in millimeters) relative to bregma were used to center the arrays: PFC (2.5–4.5 mm anterior to bragma and 0.2–1.0 mm lateral to midline, 2.5–3.0 mm deep from cortical surface). Rats were allowed 5 days to recover from the surgery.

### WM task training

**Figure 1:**
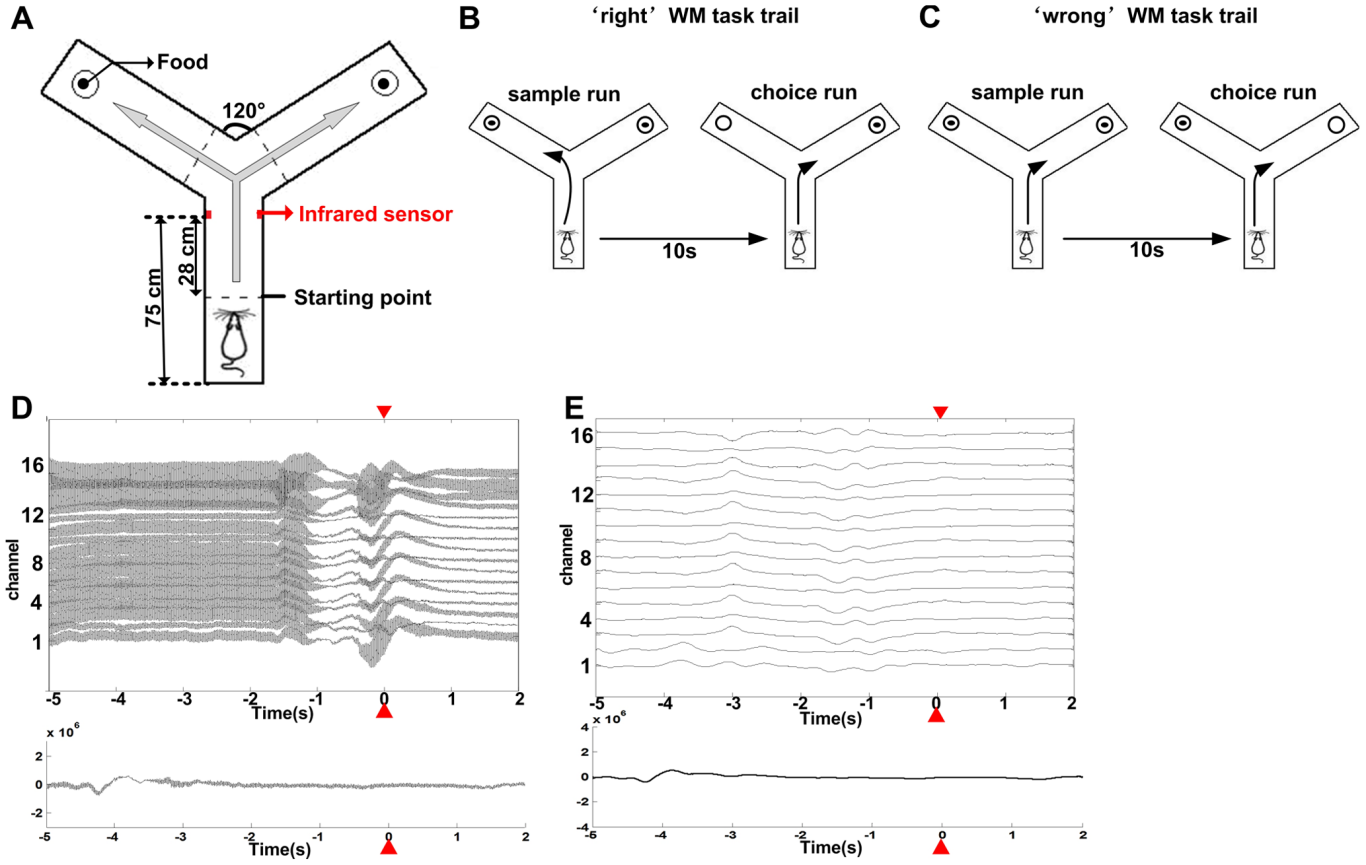
Rat working memory task training in Y-maze and one example of LFPs. (A) Y-maze for rat working memory task; (B) one example of ‘right’ task, the direction of ‘choice run’ is different to ‘sample run’; (C) one example of ‘wrong’task, the direction of ‘choice run’ is same to ‘sample run’; (D) one example of 16-channels LFPs and one channel LFPs before preprocessing; (E) one example of 16-channels LFPs and one channel LFPs after preprocessing. ▾▴ represents the tripping time by infrared in Y-maze.

Behavioral training was performed in Y-maze (**see** **Figure 1A**). Both the start arm (75 cm long) and the two arms forming the Y (both 75 cm long and diverging at a 120° angle from the stem arm) were 14.5 cm in width. The occurrence of WM task was marked by using an infrared sensor in the Y-maze and was defined as the ‘tripping point’ in the article.

The procedure was described in details by Bannerman DM [43]. Rats were subject to restrict diet and maintain at approximately 80% of their original weight and each rat was given 3 days of 30 min pre-training in order to train them to run reliably down the stem of the maze and to find peanut tablets in the food wells in both arms. They were habituated to a Y-maze until they voluntarily ate a piece of peanut placed at the end of each arm. Each WM task trial in Y-maze lasted 20–24 s and it consisted of 3 stages: (1) sample run: door at the start arm was opened and the rat was allowed to do a free choice between the two arms of the Y-maze. It might last 5–7 s; (2) rat was picked up and put back into the start arm for a delay 10 s; (3) choice run: the trial was considered ‘right’ if the rat entered the arm not previously entered on the ‘sample run’ and could be allowed to eat the food reward, if not, the trial was considered ‘wrong’ (**see** **Figure 1 B, C**). Choice run might last 5–7 s. The rats received 20 WM trials daily with each animal having one trial in turn. The correct rate was calculated every day, and when it was stable over 85%, the WM task training was ended.

### Propofol anesthesia

Rats were randomly assigned to three groups. Control rats (n = 7) received no treatment.

Remaining rats (n = 14) were anesthetized by continuous infusion propofol (AstraZenec, Italy) via a tail vein catheter. For this procedure, rats were placed in the plastic box, pro group (n = 7) receiving propofol with 0.5 mg·kg^−1^·min^−1^, 2 h and PRO group (n = 7) receiving propofol with 0.9 mg·kg^−1^·min^−1^, 2 h. All anesthetized rats were breathing spontaneously, and the body temperature of the rats was maintained at 37°±0.5°C by using a heating pad. Arterial oxygen saturation and mean arterial blood pressure were measured noninvasively using a pulse oximeter and a rat tail cuff during anesthesia (ZS-Z, ZS Dichuang, Beijing). Arterial blood samples were collected from the femoral artery at the end of propofol anesthesia and analyzed for pH, PaCO_2_, PaO_2_ (GEM, Premier 3000, Blood Gas/Electrolyte Analyzer, Model 5700) as compared with the control group.

### LFPs data acquisition and preprocessing

Wideband signals were recorded at sampling frequency 2000 Hz during rat WM task with a Cerebus data acquisition system (Cyberkinetics, Foxborough, MA). The occurrence of behavioral events was marked online by an infrared sensor in the Y-maze. 16-channel LFPs (low-pass filter 0.3–500 Hz) were extracted from each wideband channel via filters in the Neural Signal Processor. We paid particular attention to preprocessing steps given the sensitivity of directed transfer function (DTF) to standard manipulations [44], [45]. We applied two-way least-squares finite impulse response (FIR) notch filters (49–51 Hz and 99–101 Hz) to remove the 50 Hz mains-electricity line-noise as well as its harmonic at 100 Hz (**see** **Figure 1 D,E**) [46].

### Time-Frequency Analysis

After pretreatment, we investigated the time evolution of frequency power spectrum of the WM task by applying the short-time Fourier transform with a 500 ms wide Hamming window to the LFPs data. Each group had 60 sections data each day, each LFPs data had 16 channels and each channel had a spectrum, we calculated the average spectrum of 16 channels and gave a common result which can represent a group of data. In each time-frequency spectrum, peak frequency and peak power of LFPs were measured for statistical analysis. Differences in peak frequencies were evaluated by one-way analysis of variance (ANOVA). When the average peak power in one region was more than 2 times of the power in the other region, the pattern of distribution of the gamma and theta rhythms was regarded as showing clear dominance.

### DTF connectivity calculations

The directed transfer functions (DTF) method has been demonstrated to be a useful tool for the analysis of causal connections among multi-channel signals over various frequency bands [47], [48]. DTF method can transform multi-LFPs into a causal network. The nodes of the causal network are defined as the recording electrodes, and the edges of the causal network are elements of DTF matrix which describe LFPs functional connectivity directly. DTF is formulated in the framework of the multivariate autoregressive (MVAR) model [49].

In the framework of the MVAR model, multi-LFPs can be described as a data vector X of M source signals: 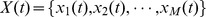



The MVAR model can then be constructed as: 
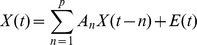
where *E (t)* represents a vector composed of white noise values at time t, *A_n_* is a M×M matrix composed of the model coefficients, and *p* is the model order. In the present study, the model order was determined by means of criteria derived using the Bayesian information criterion (BIC) [50].

The MVAR model was then transformed into the frequency domain as follows: 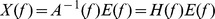
where *f* denotes a specific frequency and the *H(f)* matrix is the transfer matrix defined as: 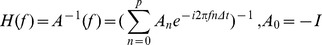
where *I* is an identity matrix.

The DTF was defined in terms of the elements of the transfer matrix *H_ij_* as: 
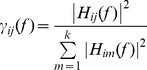
where 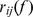 is the ratio between inflow from node *j* to node *i* and all inflows to node *i*, and *k* is the number of nodes. The functional connectivity among LFPs was calculated and converted into a DTF matrix. Therefore, the average value of DTF matrix was a direct measurement of functional connection among LFPs.

### Quantitative description of the functional connectivity

Given the causal network identified by the DTF matrix, the three calculation values: clustering coefficient (*C*), network density (*D*) and global efficiency (*E_global_*) were calculated from the elements of DTF matrix,which measured the connection features of causal network.

*Clustering coefficient*

The clustering coefficient (*C*) is a measure of the “cluster together degree” of nodes to the transit of information across the network [51], [52]. Given the causal network identified by the DTF matrix, *C* can be defined mathematically as average of the local clustering coefficient *c_i_*: 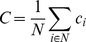


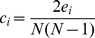
where *e_i_* is the number of nonzero element connected to node *i* in the DTF matrix. *N* is the number of nodes. V is the set of nodes in the causal network.

*Network density*

The network density (*D*) in a causal network is defined as the actual number of edges as a proportion of the total number of possible edges [53], [54]. Given the causal network identified by the DTF matrix, the *D* can be defined mathematically as: 
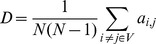
where *a_i, j_* is the value of element in the DTF matrix from node *i* to node *j*. *N* is the number of nodes. V is the set of nodes in the causal network. The higher *D* means more nodes connected to one another, which indicates the nodes in causal network were globally coordinated in their activity [55].

*Global efficiency*

The global efficiency (*E_global_*) in a causal network is defined as the inverse of the harmonic mean of path length (the path lengths were determined by taking the inverse of the strength of each DTF connection) between nodes [54], [55]. Given the causal network identified by the DTF matrix, the *E_global_* can be defined mathematically as: 
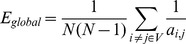
where *a_i, j_* is the value of element in the DTF matrix from node *i* to node *j*. *N* is the number of nodes. *V* is the set of nodes in the causal network. In effect, *E_global_* is a measure of the “speed” of information transfer between any pair of nodes in causal network [56], with a high value indicating a high average “speed” and efficient information transfer in a causal network [57].

### Statistical analysis

Data were presented as mean ± SEM. The D'Agostino normality test was applied before performing the ANOVA test. Differences among three groups in each day were determined by one-way ANOVA analysis followed by Newman-Keuls post hoc test. Differences across three days in each group were assessed by repeated measures one-way ANOVA and Tukey's multi-comparison test. Values of *p*<0.05 were considered statistically significant.

## Results

We measured the LFPs signal from prefrontal cortex from 21 SD rats while they were engaged in a WM task. One rat in control group, one rat in pro group and one rat in PRO group were excluded because they could not complete the task or the signals recorded exceed the span of LFP greatly. So the analysis was carried on the data from 6 control rats, 6 pro rats and 6 PRO rats.

### Effect of propofol anesthesia on WM performance

**Figure 2:**
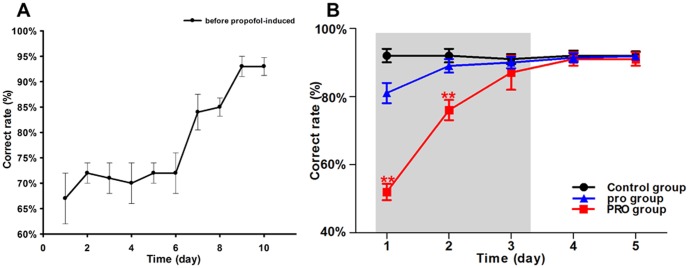
Behavioral results of rats Y-maze performance. (A) Average correct rate of SD male rats before propofol anesthesia, from the 7^th^ day, the correct rate was above 85% in three consecutive days; (B) Average correct rate after propofol anesthesia. Black line indicate control group averaged correct rate, red line indicate PRO group and blue indicate pro group. In the first 2 days, the difference is obvious between three groups, in the last 3 days, the inhibition of propofol was disappearing (** *p*<0.01). Error bars represent standard error. Control  =  no propofol anesthesia; pro = 0.5 mg•kg^−1^•min^−1^, 2 h; PRO = 0.9 mg•kg^−1^•min^−1^, 2 h.

The rats were trained to perform WM task in Y-maze before propofol-anesthesia, improvement in behavioral result was indicated by a gradual increase of correct rate. Correct rate was stable over 85% after 8 days of training (**see** **Figure 2A**). After propofol anesthesia, the correct rate of PRO group was significantly decreased when rats were performing the Y-maze WM task, however, the decrease was in the first three days shown in the gray shadow (**see** **Figure 2B**). We could not detect significant difference of WM performance among groups during the forth and fifth days by using ANOVA one-way post hoc Newman-Keuls test.

### Time-frequency analysis of LFPs

**Figure 3:**
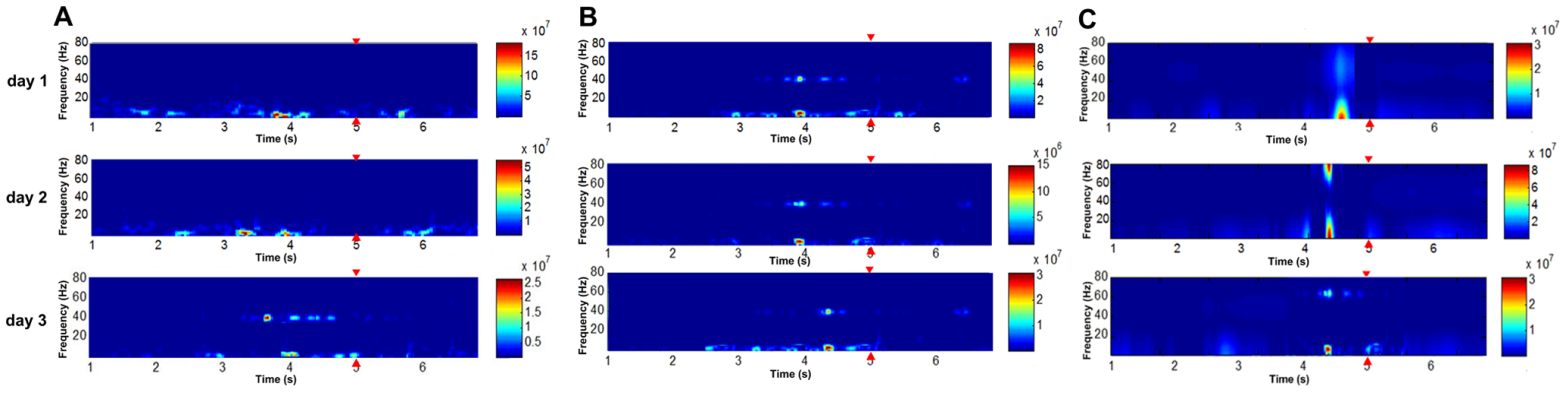
Time-frequency spectra of LFPs while rat in a WM task. Spectral peaks of the gamma and theta rhythms stand out as isolated spots (arrows). The gamma rhythms slow down gradually from 55.0 Hz and 75.0 Hz while theta rhythms slow down gradually from 3.0 Hz and 10.0 Hz. The time interval of LFPs was 2 s before WM reference and 1 s after WM reference. Time is presented on the x-axis. Frequency is presented on the y-axis. The width of each spectral segment was 7 s, and the frequency ranges was 1–80 Hz. (A) denote time-frequency spectra of PRO group in the first day, the second day and the next day after propofol anesthesia. (B), (C) respectively denote time-frequency spectra of control group and pro group at the same time. The energy level is coded on a color scale: blue areas show low energy, and red areas show high energy. Control  =  no propofol anesthesia; pro = 0.5 mg•kg^−1^•min^−1^, 2 h; PRO = 0.9 mg•kg^−1^•min^−1^, 2 h. ▾▴ represents the tripping time by infrared in Y-maze.

After preprocessing, 60 sections data of each group each day were picked to compute the time-frequency spectrum. In each section, LFPs data had 16 channels and each channel had a spectrum. We calculated the average spectrum of 16 channels and showed a common result which could represent a group of data (**see** **Figure 3**).

### Effects of propofol anesthesia on LFPs functional connectivity

Time-frequency spectra had showed the gamma and theta rhythms of LFPs played a key role in WM task. We used band-pass filter to obtain gamma and theta band LFPs. DTF method was applied to LFPs to obtain a DTF matrix which described the strength of LFPs functional connectivity directly. Average value of DTF matrix, clustering coefficient (*C*), network density (*D*) and global efficiency (*E_global_*) were computed to describe the functional connectivity quantitatively.

#### Average value of DTF matrix

**Figure 4:**
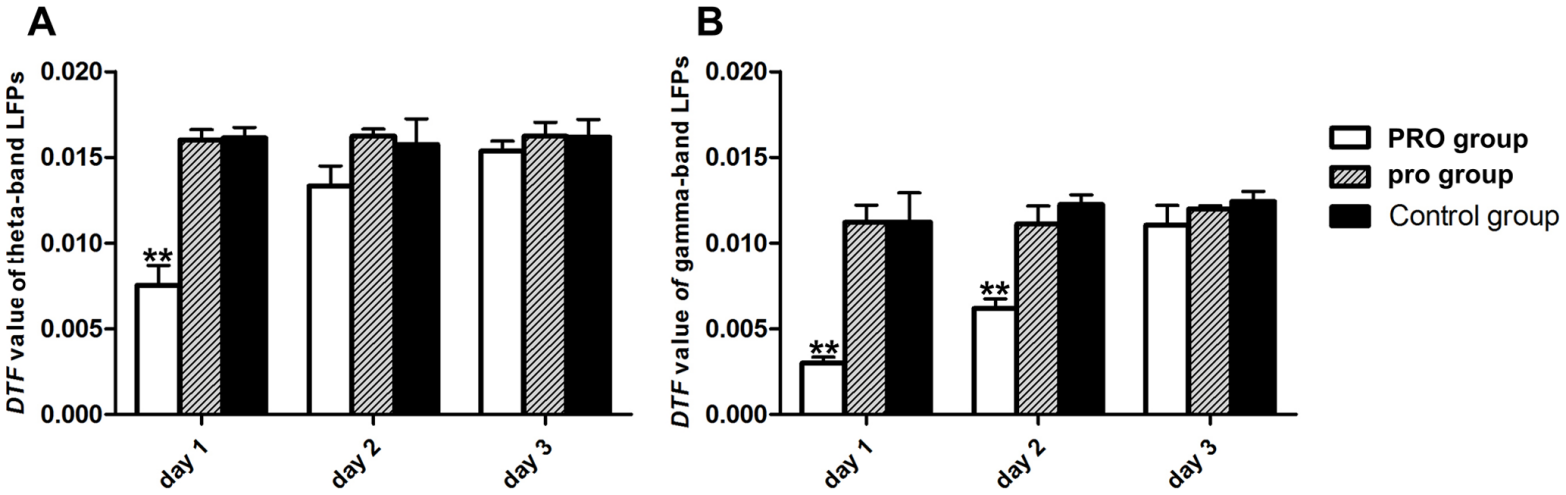
The average value of DTF matrix of three groups. (A) Theta-band LFPs; (B) Gamma-band LFPs. Error bars represent standard error. * *p*<0.05, ** *p*<0.01, ANOVA one-way post hoc Newman–Keuls test. Control  =  no propofol anesthesia; pro = 0.5 mg•kg^−1^•min^−1^, 2 h; PRO = 0.9 mg•kg^−1^•min^−1^, 2 h.

In gamma-band LFPs causal network, average value of DTF matrix of PRO group was significantly decreased in the first 2 days, and no significant difference was found in the 3^rd^ day. In theta-band LFPs causal network, average value of DTF matrix of PRO group was significantly decreased only in the 1^st^ day (see Figure 4, Table 1), which meant propofol anesthesia with 0.9 mg·kg^−1^·min^−1^, 2 h could weaken the functional connectivity among LFPs causal network in the first 2 days.

**Table 1:** Changes in average value of DTF matrix of three groups in three days.

	Theta-band LFPs			Gamma-band LFPs		
DTF value	PRO	pro	Control	PRO	pro	Control
**Day 1**	0.0082±0.0021**	0.0161±0.0014	0.0162±0.0012	0.0032±0.0011**	0.0119±0.0017	0.0119±0.0028
**Day 2**	0.0140±0.0023	0.0168±0.0013	0.0165±0.0019	0.0065±0.0013**	0.0119±0.0019	0.0122±0.0013
**Day 3**	0.0157±0.0029	0.0166±0.0011	0.0165±0.0014	0.0117±0.0024	0.0121±0.0009	0.0121±0.0015

Results are expressed as mean ± SEM of the whole group (n = 6).

*p<0.05,

**p<0.01, ANOVA one-way post hoc Newman–Keuls test. Control  =  no propofol anesthesia; pro = 0.5 mg•kg^−1^•min^−1^, 2 h; PRO = 0.9 mg•kg^−1^•min^−1^, 2 h.

We also investigated the differences in each group in three days after anesthesia by one-way ANOVA. The average value of DTF matrix of PRO group were significant increased from 0.0082±0.0021 at the first day to 0.0157±0.0029 at the third day in theta-band LFPs causal network (*F*_0.05 (2, 177)_ = 3.84, *p*<0.05) and from 0.0032±0.0011 at the first day to 0.0117±0.0024 at the third day in gamma-band LFPs causal network (*F*_0.01 (2, 177)_ = 5.26, *p*<0.01). The average value of DTF matrix of pro group (theta: *F*_0.05 (2, 177)_ = 0.94, *p*>0.05; gamma: *F*_0.05 (2, 177)_ = 1.17, *p*>0.05) and control group (theta: *F*_0.05 (2, 177)_ = 1.22, *p*>0.05; gamma: *F*_0.05 (2, 177)_ = 0.81, *p*>0.05) remained stable.

#### Clustering coefficient

**Figure 5:**
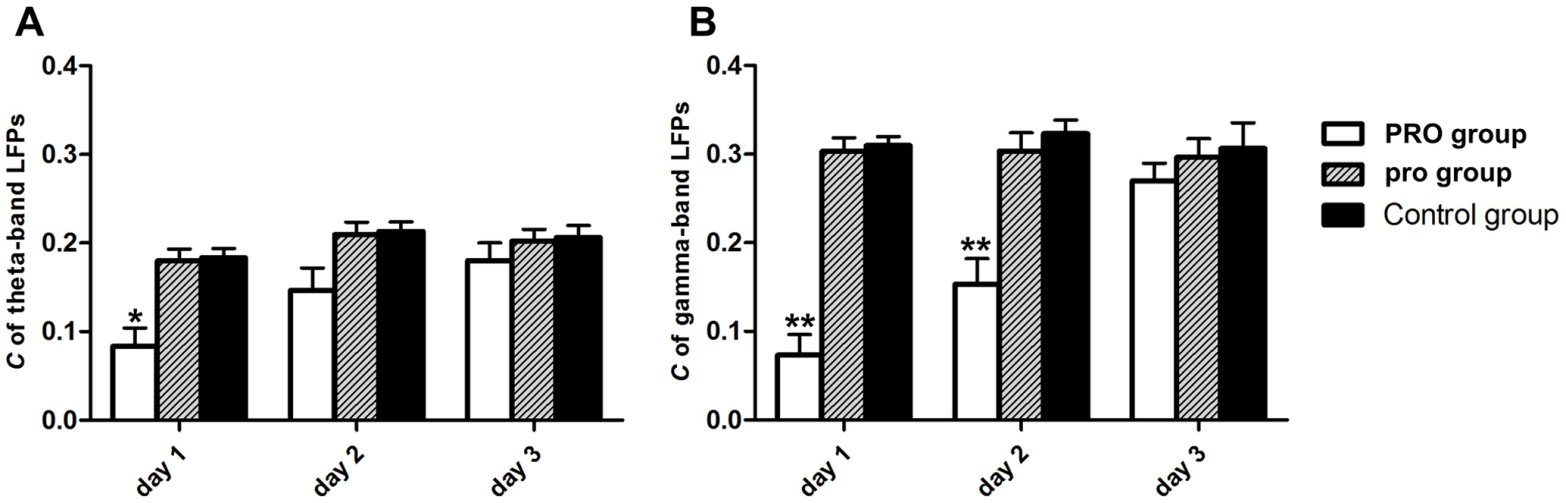
Clustering coefficient of LFPs functional connectivity across three groups. (A) Theta-band LFPs; (B) Gamma-band LFPs. Error bars represent standard error. * *p*<0.05, ** *p*<0.01, ANOVA one-way post hoc Newman–Keuls test. Control  =  no propofol anesthesia; pro = 0.5 mg•kg^−1^•min^−1^, 2 h; PRO = 0.9 mg•kg^−1^•min^−1^, 2 h.

In gamma-band LFPs causal network, *C* of PRO group was significantly decreased in the first 2 days after anesthesia, and no significant difference was found in the 3^rd^ day; in theta-band LFPs causal network, *C* of PRO group was significantly decreased only in the 1^st^ day (see Figure 5, Table 2), which meant the connection of nodes in LFPs causal network was more random in PRO group. Propofol anesthesia with 0.9 mg·kg^−1^·min^−1^, 2 h could damage to the regularity of functional connectivity in LFPs causal network in the first 2 days.

**Table 2:** Changes in clustering coefficient of three groups in three days.

	Theta-band LFPs			Gamma-band LFPs		
*C*	PRO	pro	Control	PRO	pro	Control
**Day 1**	0.09±0.03*	0.17±0.02	0.18±0.05	0.06±0.03**	0.30±0.02	0.31±0.03
**Day 2**	0.15±0.03	0.20±0.03	0.21±0.05	0.17±0.04**	0.30±0.02	0.32±0.04
**Day 3**	0.16±0.02	0.19±0.02	0.20±0.03	0.27±0.03	0.28±0.03	0.29±0.05

Results are expressed as mean ± SEM of the whole group (n = 6).

*p<0.05,

**p<0.01, ANOVA one-way post hoc Newman–Keuls test. Control  =  no propofol anesthesia; pro = 0.5 mg•kg^−1^•min^−1^, 2 h; PRO = 0.9 mg•kg^−1^•min^−1^, 2 h.

We investigated the differences in each group in three days by one-way ANOVA. *C* of PRO group were significant increased from 0.09±0.03 at the first day to 0.16±0.02 at the third day in theta-band LFPs causal network (*F*_0.05 (2, 177)_ = 3.11, *p*<0.05) and from 0.06±0.03 at the first day to 0.27±0.03 at the third day in gamma-band LFPs causal network (*F*_0.01 (2, 177)_ = 7.06, *p*<0.01), while *C* of pro group (theta: *F*_0.05 (2, 177)_ = 1.93, *p*>0.05; gamma: *F*_0.05 (2, 177)_ = 1.82, *p*>0.05) and control group remained stable (theta: *F*_0.05 (2, 177)_ = 2.12, *p*>0.05; gamma: *F*_0.05 (2, 177)_ = 1.08, *p*>0.05).

#### Network density

**Figure 6:**
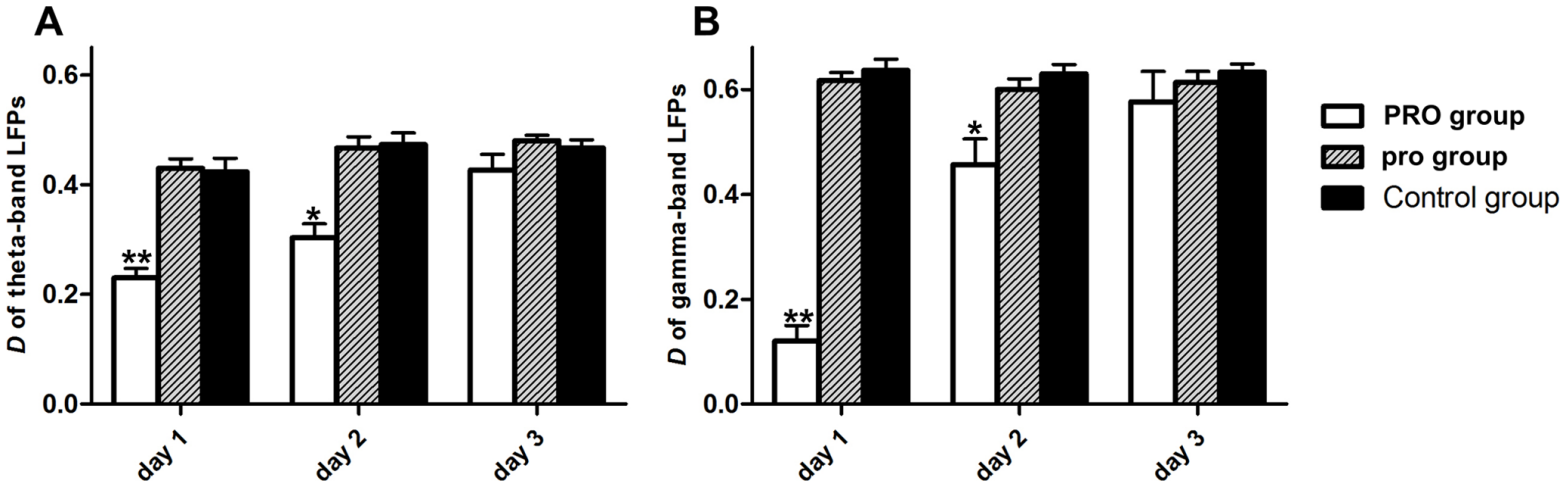
Network density of LFPs functional connectivity across three groups. (A) Theta-band LFPs; (B) Gamma-band LFPs. Error bars represent standard error. * *p*<0.05, ** *p*<0.01, ANOVA one-way post hoc Newman–Keuls test. Control  =  no propofol anesthesia; pro = 0.5 mg•kg^−1^•min^−1^, 2 h; PRO = 0.9 mg•kg^−1^•min^−1^, 2 h.

D of PRO group was significantly decreased in the first 2 days, and no significant difference was found in the 3^rd^ day (see Figure 6, Table 3), which meant the connection of nodes in LFPs causal network was sparser in PRO group. Propofol anesthesia with 0.9 mg·kg^−1^·min^−1^, 2 h could damage the denseness of functional connectivity in LFPs causal network in the first 2 days.

**Table 3:** Changes in network density of three groups in three days.

	Theta-band LFPs			Gamma-band LFPs		
*D*	PRO	pro	Control	PRO	pro	Control
**Day 1**	0.22±0.03**	0.42±0.02	0.42±0.07	0.12±0.03**	0.61±0.03	0.63±0.02
**Day 2**	0.30±0.04*	0.48±0.03	0.48±0.06	0.48±0.04*	0.59±0.04	0.62±0.03
**Day 3**	0.41±0.02	0.48±0.02	0.47±0.05	0.61±0.05	0.61±0.04	0.63±0.04

Results are expressed as mean ± SEM of the whole group (n = 6).

*p<0.05,

**p<0.01, ANOVA one-way post hoc Newman–Keuls test. Control  =  no propofol anesthesia; pro = 0.5 mg•kg^−1^•min^−1^, 2 h; PRO = 0.9 mg•kg^−1^•min^−1^, 2 h.

We also investigated the differences in each group in three days by one-way ANOVA. *D* of PRO group was significantly decreased of propofol group from 0.22±0.03 at the first day to 0.41±0.02 at the third day in theta-band LFPs causal network (*F*_0.05 (2, 177)_ = 3.73, *p*<0.05) and from 0.12±0.03 at the first day to 0.61±0.05 at the third day in gamma-band LFPs causal network (*F*_0.01 (2, 177)_ = 5.73, *p*<0.01). *D* of pro group (theta: *F*_0.05 (2, 177)_ = 1.36, *p*>0.05; gamma: *F*_0.05 (2, 177)_ = 1.07, *p*>0.05) and control group remained stable (theta: *F*_0.05 (2, 177)_ = 1.53, *p*>0.05; gamma: *F*_0.05 (2, 177)_ = 0.73, *p*>0.05).

#### Global efficiency

**Figure 7:**
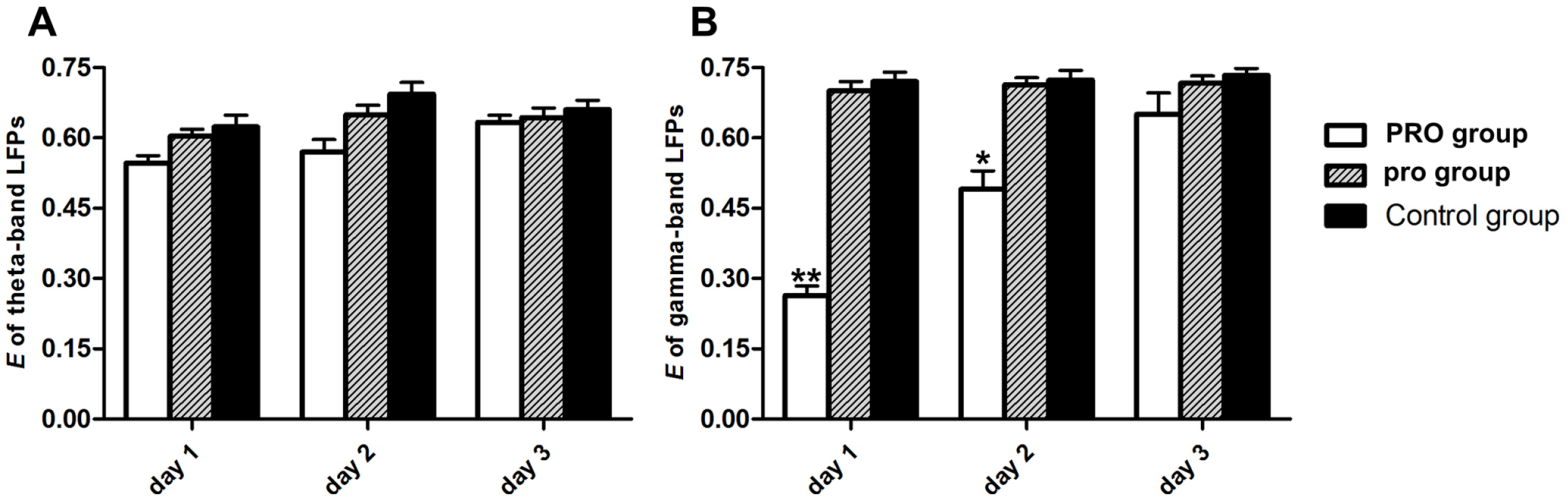
Global efficiency of LFPs functional connectivity across three groups. (A) Theta-band LFPs; (B) Gamma-band LFPs. Error bars represent standard error. * *p*<0.05, ** *p*<0.01, ANOVA one-way post hoc Newman–Keuls test. Control  =  no propofol anesthesia; pro = 0.5 mg•kg^−1^•min^−1^, 2 h; PRO = 0.9 mg•kg^−1^•min^−1^, 2 h.

Only in gamma-band LFPs causal network, E_global_ of PRO group was significantly decreased in the first 2 days, but no significant difference was found in the 3^rd^ day, neither in theta-band LFPs causal network in the first 3 days (see Figure 7, Table 4). Propofol anesthesia with 0.9 mg·kg^−1^·min^−1^, 2 h could decrease the efficiency of parallel information transfer only in gamma-band LFPs causal network in the first 2 days.

**Table 4:** Changes in global efficiency of three groups in three days.

	Theta-band LFPs			Gamma-band LFPs		
*E_global_*	PRO	pro	Control	PRO	pro	Control
**Day 1**	0.57±0.01	0.60±0.02	0.62±0.04	0.24±0.02**	0.70±0.03	0.72±0.02
**Day 2**	0.58±0.03	0.63±0.03	0.67±0.02	0.49±0.05*	0.71±0.01	0.73±0.01
**Day 3**	0.62±0.02	0.63±0.02	0.66±0.01	0.69±0.04	0.72±0.01	0.73±0.01

Results are expressed as mean ± SEM of the whole group (n = 6).

*p<0.05,

**p<0.01, ANOVA one-way post hoc Newman–Keuls test. Control  =  no propofol anesthesia; pro = 0.5 mg•kg^−1^•min^−1^, 2 h; PRO = 0.9 mg•kg^−1^•min^−1^, 2 h.

We investigated the differences in each group in three days by one-way ANOVA. *E_global_* of PRO group were significant increased from 0.24±0.02 at the first day to 0.69±0.04 at the third day in gamma-band LFPs causal network (*F*_0.01 (2, 177)_ = 4.97, *p*<0.01) and from 0.57±0.01 at the first day to 0.62±0.02 at the third day in theta-band LFPs causal network (*F*_0.05 (2, 177)_ = 2.81, *p*>0.05). *E_global_* of pro group (theta: *F*_0.05 (2, 177)_ = 1.34, *p*>0.05; gamma: *F*_0.05 (2, 177)_ = 0.75, *p*>0.05) and control group remained stable (theta: *F*_0.05 (2, 177)_ = 1.78, *p*>0.05; gamma: *F*_0.05 (2, 177)_ = 0.53, *p*>0.05).

## Discussion

We investigated the inhibition mechanism of propofol anesthesia on working memory function from the view of functional connectivity. The LFPs causal network was defined by DTF method which measured the LFPs functional connection directly. The three calculation values: clustering coefficient (*C*), network density (*D*) and global efficiency (*E_global_*) were calculated from the elements of DTF matrix,which describe the connection features of causal network. The main findings are: (1) Propofol anesthesia with 0.5 mg•kg^−1^•min^−1^, 2 h does not inhibit rats WM function. (2) A prominent inhibition on rats WM function is induced by propofol anesthesia with 0.9 mg•kg^−1^•min^−1^,2 h, but the inhibition dose lasts less than 72 hours; which may mainly inhibit the functional connectivity of gamma-band LFPs which play a key role in WM task and eventually lead to memory dysfunction. (3) Network-based analyses of task-based functional connectivity analysis of LFPs can be a useful tool for delineating the effect of propofol anesthesia on working memory function.

### The vital signs and arterial blood gas during propofol anesthesia

The blood pressure, blood gases, and PH values in 2 propofol groups were within the normal physiologic range and no significant difference was found compared with the control group (**see** **Table 5**). Taking these measures reduces the possibility that propofol-induced affect learning and memory function of the brain was caused by physiologic side effects such as hypoglycemia, hypoxia and hypercapnia.

**Table 5:** Blood pressure, blood gases, and PH measured in all groups.

	MAP (mmHg)	SaO_2_ (%)	PH	PaCO_2_ (mmHg)	PaO_2_ (mmHg)
**pro group**	95.5±4.3	97.3±2.4	7.35±0.03	41.1±0.87	160.5±7.42
**PRO group**	93.2±8.9	96.2±1.1	7.41±0.01	40.8±1.21	165.2±4.11
**Control group**	94.1±8.8	95.9±1.2	7.39±0.02	41.2±1.62	156.9±5.25

Values are mean ± SEM. MAP  =  mean arterial blood pressure; SaO_2_  =  arterial oxygen saturation; PaCO_2_  =  arterial carbon dioxide tension; PaO_2_  =  arterial oxygen tension. Control  =  no propofol anesthesia; pro = 0.5 mg•kg^−1^•min^−1^, 2 h; PRO = 0.9 mg•kg^−1^•min^−1^, 2 h.

### The DTF method as a tool for estimating LFPs causal connectivity

Franaszczuk et al. first applied the DTF technique to EEG data acquired from patients with epilepsy and demonstrated that the patterns of seizure propagation could be identified successfully using DTF analysis [58], which suggested that the DTF-based analysis of networks is a powerful technique and the DTF values could describe the strength of functional connection among LFPs directly. Since that study, Anna Korzeniewska et al. analyzed the direct information transfer among brain structures on the basis of LFP, and it demonstrated that DTF method could be used to obtain the reliable patterns of connections between various brain structures [59]. Recently, Christopher Wilke and Bin He et al. have applied the graph theory methods and DTF connectivity analysis to ECoG data acquired from patients undergoing presurgical monitoring for the treatment of epilepsy and compared network causal interactions during different time points and frequency bands [60]. Furthermore, Brzezicka A analyzed the WM task EEG by means of the DTF method and found stronger transmissions in theta and alpha band accompanied by flows in gamma band of EEG [61], which was consistent with previous fMRI data concerning fronto-parietal regions involvement in working memory processes [62], [63]. Their results provide evidence suggesting that the DTF method is reliable and accurate.

### Propofol mainly inhibit gamma-band LFPs functional connectivity

As a preliminary step of the LFPs data analysis, we evaluated the spectral power of LFPs rhythms in propofol and control groups, in line with the vast majority of previous field studies. Spectral power of LFPs showed the theta, gamma rhythms over wide regions of the scalp was characterized by a marked power decrease in the first 2 days after propofol anesthesia. Therefore we were more concerned about the altering in theta-band and gamma-band LFPs functional connectivity.

The results showed that the averaged DTF value of gamma-band LFPs in the propofol group was decreased significantly in 24 h and 48 h after propofol anesthesia, but no differences in theta-band LFPs at 48 h. This implied that propofol anesthesia was mainly influence on gamma-band of LFPs during WM task. Furthermore, *C*, *D* and *E_global_* in gamma-band LFPs causal network of propofol group were significantly decreased in 48 h after propofol anesthesia, therefore, the network of gamma-band LFPs was random, sparser, and low efficiency while brain over which the brain processes information in 48 h after propofol anesthesia. The results indicate that propofol mainly inhibit the gamma-band LFPs oscillation, which are in line with previous research that gamma rhythms LFPs in PFC could be important for memory replay [64]–[68].

Propofol, potentiating GABA_A_ receptor activity, can block the gamma rhythm induced by electrical and chemical stimulation, and regulation of gamma rhythm translation by adjusting the opening of the GABA_A_ ligand-gated channels [69], which might induce protein molecule changes in the membrane of cortical cells so as to modify their contacts and thus reshape their functional connectivity networks.

### Inhibition of propofol anesthesia is not unrecoverable

**Figure 8:**
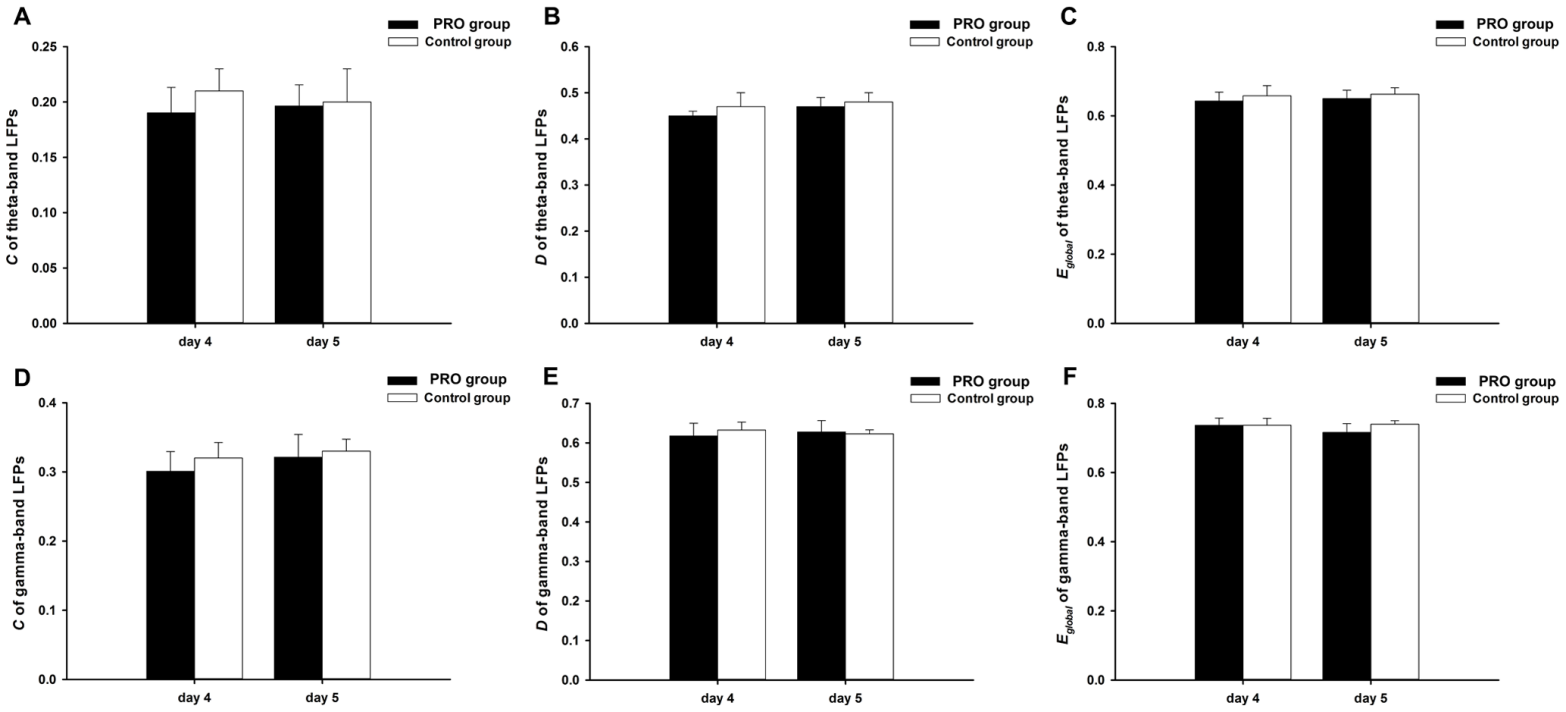
Quantitative description of LFPs functional connectivity across PRO group (black) and control group (white) in the 4th day and 5th day. (A), (B) and (C) respectively denote *C*, *D* and *E_global_* of theta-band LFPs causal network; (D), (E) and (F) respectively denote *C*, *D* and *E_global_* of theta-band LFPs causal network. Error bars represent standard error. Control  =  no propofol anesthesia; PRO = 0.9 mg•kg^−1^•min^−1^, 2 h.

This study also demonstrated that the inhibition of propofol anesthesia on WM was not irreversible in rats. It was in line with previous studies that psychomotor and memory function are impaired for only 24 h–48 h or less after surgery under propofol anesthesia [70]–[72]. We recorded LFPs in PFC during rat WM task in Y-maze and investigated network properties such as *C*, *D* and *E_global_* of PRO and control group on the 4^th^ and 5^th^ day after propofol-induced (**see** **Figure 8**) and did not found significant difference.

In summary, our results suggest that the effects of propofol on rats WM function are dose dependent, gamma-band LFPs in PFC plays a central role in the coordinated reactivation of stored memories, which directly affects the correct rate of rat WM task in Y-maze. The strength of the energy and the efficiency of information transform among gamma-band LFPs network can be inhibited in 72 h after propofol anesthesia with 0.9 mg·kg^−1^·min^−1^, 2 h, but the inhibition is not unrecoverable. Besides, network-based analyses of task-based functional connectivity may provide a new insight about the neurophysiology mechanism of memory impairment induced by propofol.
